# Glycemic Control Outcomes by Gender in the Pay-for-Performance System: A Retrospective Database Analysis in Patients with Type 2 Diabetes Mellitus

**DOI:** 10.1155/2014/575124

**Published:** 2014-08-18

**Authors:** Shao-Ping Yuan, Chien-Ning Huang, Hung-Chang Liao, Yu-Tzu Lin, Ya-huei Wang

**Affiliations:** ^1^Institute of Medicine, Chung Shan Medical University, Department of Medical Management, Chung Shan Medical University Hospital, No. 110, Section 1, Jianguo N. Road, Taichung City 40201, Taiwan; ^2^Institute of Medicine, Chung Shan Medical University, Department of Endocrinology & Metabolism, Chung Shan Medical University Hospital, No. 110, Section 1, Jianguo N. Road, Taichung City 40201, Taiwan; ^3^Department of Health Services Administration, Chung Shan Medical University, Department of Medical Education, Chung Shan Medical University Hospital, No. 110, Section 1, Jianguo N. Road, Taichung City 40201, Taiwan; ^4^Diabetes Education Center, Chung Shan Medical University Hospital, No. 110, Section 1, Jianguo N. Road, Taichung City 40201, Taiwan; ^5^Department of Applied Foreign Languages, Chung Shan Medical University, Department of Medical Education, Chung Shan Medical University Hospital, No. 110, Section 1, Jianguo N. Road, Taichung City 40201, Taiwan

## Abstract

*Background*. The purpose of this study was to investigate how the degree of glycemic control in patients with type 2 diabetes associated with lifestyle interventions as well as sociodemographic factors and further examine the differences by gender. *Methods*. This was a retrospective study using data collected from a diabetes quality improvement plan that began in 2002 in a medical center in Taiwan. Statistic analysis was used to determine the associations of sociodemographic data, lifestyle intervention, and treatment regimens with changes in HbA1c levels (between the initial visit and the latest follow-up measured level), and the differences were then sorted by the sex of the patients. *Results*. Our results showed that HbA1c averaged 7.50% for males and 7.80% for females at the initial visit, compared to levels averaging 7.50% for males and 7.70% for females at the most recent follow-up visit. There was no significant change (*P* = 0.541) in HbA1c in males, but there was a 0.10% (*P* = 0.384) reduction in females. The duration of the diabetes and medication regimen was associated with the decrease seen in the females. *Conclusions*. The results of these analyses provide important insights for policy makers to formulate healthcare policies related to chronic diseases or illnesses.

## 1. Background

Diabetes mellitus occurs throughout the world. Globally, as of 2012, an estimated 346 million people have type 2 diabetes [1]. The greatest increase in prevalence, however, is expected to occur in Asia and Africa, where it is estimated that the number of patients diagnosed with diabetes in the year 2000 will double by 2030 [2]. Patients with type 2 diabetes have a higher risk of developing chronic disability. A recent study suggests that diabetes is independently associated with disability [3].

As of 2012, there are approximately 1.35 million people with the disease in Taiwan; type 2 diabetes makes up about 98% of the cases of diabetes, the other 2% being due to diabetes mellitus type 1 and gestational diabetes. Data from the Department of Health, Executive Yuan, Taiwan, showed that in 2009, the prevalence of diabetes in Taiwan was estimated to be 9.2%; the prevalences found in the 1993–1996 and 2005–2008 surveys were 5.33% and 9.05%, respectively [4]. As of 2011, diabetes was the sixth leading cause of death for males and the third leading cause of death for females on Taiwanese death certificates [5]. It is well known that effective management of diabetes is important not only in terms of quality of life and in life expectancy, but also economically. Diabetes care has been shown to treat the many complications that can result from the disease itself and from its treatment, and those living with the disease devote approximately 3/4 of their medical expenditure directly to diabetic medication and associated services and materials. In 2011, the yearly direct medical cost of diabetes was over $54 billion NT (NT$30 = approx. US$1), which equals 11.5% of the total healthcare resources in Taiwan [6]. The increasing prevalence of diabetes poses both clinical and public health challenges for healthcare systems and governments.

Based on the concept of “buying health,” the Bureau of National Health Insurance (NHI, social insurance in Taiwan, a single-payer system run by the government), Executive Yuan, Taiwan, selected diabetes (along with a few other common diseases) to be handled under “pay-for-performance” plans implemented in August 2006. This scheme was amended from the Diabetes Quality-Based Payment Initiative, which started in November 2001 [7]. It is hoped that by improving the quality and efficiency of health care, the basis of the payment method (based on the patient's progress rather than individual visits), and appropriate incentives, healthcare institutions will provide patients with comprehensive and continuous care. Also, concerning physician and institutional remuneration, it is hoped that physician remuneration based on treatment targets met or patient outcomes achieved rather than an activity based system may lead to improved healthcare efficiencies. Study has shown that after the introduction of pay-for-performance program for diabetes care in Taiwan, patients enrolled in the program received more diabetes-specific exams and two more physician visits than those in the comparison group. Though they had to pay higher expenses for exams and physician visits, their expenses for inpatient services and hospitalization were significantly lowered [8, 9]. The integrity of the diabetes care project, which coordinates nurses, dieticians, and physicians, has been promoted for over a decade in Taiwan, and improvements have been made in the care of patients with type 2 diabetes [8].

Many studies have shown that intervention, including screening, diagnostic, and therapeutic actions, can be an effective way of improving glycemic control and increasing performance in relevant laboratory tests in the short term. However, data on the long-term quality of diabetes care are lacking. Numerous studies have shown the chronic care model to be an effective framework for improving the quality of diabetes care [10]. Self-management support is one of six core elements included in the chronic care model. The purpose of our study was to investigate how the degree of glycemic control in patients with type 2 diabetes associates with lifestyle interventions as well as sociodemographic factors and further examine the differences by gender. We also explored the importance of acknowledging the type of treatment. In addition, we analyzed whether inequalities in health status and disease control existed between genders.

## 2. Methods

### 2.1. Data Registry

In this retrospective study, we used the research database of the diabetic shared-care network, a hospital-based outpatient case management registry maintained by Chung Shan Medical University Hospital, Taiwan (CSMUH). CSMUH is an academic medical center with 1,304 licensed beds; it is a member hospital of the National Health Insurance diabetes mellitus medical reimbursement improvement program and was also incorporated into the Taichung City, Taiwan, diabetic shared-care network in 2002. A sample of 2,022 eligible participants, who were diagnosed with type 2 diabetes mellitus according to the classification of the International Classification of Diseases, 9th Revision, Clinical Modification codes 250.XX, and had taken part in the CSMUH diabetes program between June 2002 and February 2012, was included. Those diagnosed with non-type 2 diabetes mellitus were excluded from the study. All included diabetes patients, male and female, were above 20 years old and of Taiwanese ethnic origin as declared by the subjects. The components of the comprehensive diabetes evaluation were established in the registry and referred to the Clinical Practice Guidelines for Diabetes published by the Diabetes Association of the Republic of China. The study was approved by the Institutional Review Board of CSMUH.

### 2.2. Data Analysis

A multilevel analysis of the effects of active patient involvement in diabetes self-management tasks was undertaken in this study. Clinical and sociodemographic data were explored, including diabetes self-management health education records. Content analyses were conducted on the goals selected, and descriptive analyses were conducted on the intervention satisfaction questions. After expert panel discussions, they came to an agreement that some patient characteristics having nothing to do with the glycemic control were not included in the study, such as height, country of birth, and date of birth. The chosen patient characteristics were listed and analyzed to investigate how the degree of glycemic control in patients with type 2 diabetes associates with the chosen characteristics. The characteristics used in the study data were as follows: age, gender, education level, tobacco use, alcohol use, years of CSMUH care, duration of diabetes, family history of diabetes, hemoglobin A1c (HbA1c), body mass index (BMI), and diabetes therapy. The major features of lifestyle interventions in the self-management education records that we elicited information on were (i) physical activity; (ii) self-monitoring of blood glucose; (iii) medication compliance; and (iv) willingness to comply or undertake self-management. As planned, we completed two rounds of data collection (the initial visit and the most recent clinic visit).

For the period from June 2002 to February 2012, all of the patients who had been under CSMUH care (routine care, visiting an endocrinologist every three months) were divided into follow-up periods of 0–5 and 6–10 years. A lower HbA1c level indicates good long-term glucose control; therefore, we set the HbA1c level as a surrogate marker of metabolic control. Patients were grouped into those who achieved good blood glucose control with levels < 6.5% (according to HbA1c goals suggested by the American Diabetes Association (ADA)) [11]; those who achieved good control with levels 6.5–<7.0%; those who achieved standard glycemic control with levels 7.0–8.0%; and those who achieved poor glycemic control with levels > 8.0%. We used a multiple linear regression model in which the HbA1c level at the initial visit was the independent variable of primary interest, and the dependent variable was the HbA1c level measured at the latest follow-up. We also included age, years of CSMUH care, duration of diabetes, education, family history, BMI, physical activity, tobacco use, alcohol use, SMBG, medication compliance, willingness to undertake self-management, and medication regimen as additional independent variables to assess whether they were potential determinants. We then used a multiple linear regression analysis to estimate the association between the risk factor and the outcome adjusting for those determinants. The test of the significance of the regression coefficient associated with the risk factor was used to assess whether the association between risk factors is statistically significant after accounting for one or more determinant variables.

Data were analyzed using SPSS software (version 14.0). Continuous variables were tested for deviation from the normal distribution, and means and SDs were calculated; for distributions that were not normally distributed, median and interquartile range were calculated instead. Nominal variables were described using frequency counts (%). Associations between nominal variables were assessed using the Chi-square test (*χ* ^2^). Differences according to the gender of the patient were assessed using *χ* ^2^ for categorical variables. A significant level of *P* < 0.05 was considered statistically significant for all analyses.

## 3. Results

### 3.1. Patient Characteristics

The average age of the female patients was greater than that of the male patients in the CSMUH diabetes program, at 64.15 years versus 61.07 years, respectively (see Table 1). The median age at diagnosis with type 2 diabetes for the male patients was 51.42 ± 12.04 (group average); it was 51.48 ± 12.17 for patients seen at the CSMUH diabetes unit for 0–5 years and 51.05 ± 11.17 for those patients treated for 6–10 years. For the female patients, the median age at diagnosis was 53.37 ± 11.26 (group average), 53.63 ± 11.48 for patients treated under the program for 0–5 years, and 51.74 ± 9.70 in the 6–10 years treatment period (see Table 3).

In this study, the average duration of diabetes was 10.91 years for all patients, 10.34 years for men and 11.48 years for women. The average number of years for which the diabetes patients received care from CSMUH was 4.4 years for all patients, 4.28 years for males and 4.52 years for females (see Table 1). The 39.5% (*n* = 398) of female patients and 30.1% (*n* = 301) of male patients in the longer diabetes duration group (>10 years) had higher HbA1c levels (7.0–8.0%, >8.0%). In all, 34.9% (*n* = 349) of the male patients and 27.5% (*n* = 281) of the female patients achieved the target level of <7.0% recommended by the ADA for nonpregnant adults (Table 2).

In education level, there was no difference by gender in the distribution subjects stratified by HbA1c levels and education level. Females were less well educated overall in this study population in both genders. The 56.8% (*n* = 528) of male patients while 79.4% (*n* = 750) of female patients had an education level under or equivalent to junior high school.

As for higher body mass index (BMI ≥ 25 kg/m^2^), of 436 male patients, 63.8% (*n* = 278) was out of the target range (HbA1c < 7.0%), while of 377 female patients, 71.3% (*n* = 269) had a high HbA1c level (HbA1c ≥ 7.0%; see Table 2). However, the BMI levels were associated with higher HbA1c levels in males (*P* = 0.038) but not in females (*P* = 0.520; see Table 2).

Regarding having SMBG or not and the degree of glycemic control, the results showed that in either male or female patients, having SMBG (once/daily or once/weekly) has no association with the degree of glycemic control.

### 3.2. Relationship between Diabetes Care and Self-Management

Diabetes care and self-management had the largest impact on patients' own treatment goals. The patients in this study were not physically active at baseline, and only 37.8% of males (*P* = 0.003) and 39.4% of females (*P* = 0.052) reported achieving an exercise goal of 150 minutes per week at the most recent follow-up. Regarding physical activities, of 378 male patients saying having exercises for 150 minutes weekly, 61.4% (*n* = 232) had a high HbA1c level (HbA1c ≥ 7.0%); of 403 female patients, 69.7% (*n* = 281) had a high HbA1c level (see Table 2). As for the association between having physical activities and the degree of glycemic control, having physical activities (150 min/weekly) is more associated with the degree of glycemic control in males (*P* = 0.003) than in females (*P* = 0.052; see Table 2). An examination of smoking and excessive alcohol intake interactions showed that male patients consuming alcohol were more likely to have an HbA1c level ≥ 7.0% (a total of 43.9%, *n* = 412). Regarding self-monitoring of blood glucose (SMBG) among male patients, 51.3% (*n* = 453) were monitored at least once per week, and 5.2% (*n* = 52) were monitored at least once per day. Of the female patients, 46.2% (*n* = 431) were monitored at least once per week and 5.6% (*n* = 57) were monitored at least once per day.

There was no association found between adhering to one's medication regimen and HbA1c level among men, whereas among women, adhering to prescribed medication regimen was associated with having a lower HbA1c. The findings showed that 49% of those with HbA1c > 8.0% partially adhered to their prescribed medication regimen, compared to 37.2% of those with HbA1c < 7.0% (*P* = 0.038).

Patient willingness to perform self-care as intended was classified into two major categories: strong willingness and average willingness. A total of 82.6% of the male patients expressed strong willingness to perform self-care, compared to 84.4% of the female patients. The results showed that 62.1% of the male patients (402 out of 647) and 69.3% of the female patients (480 out of 693) in the strong willingness group had poor glycemic control, and 68.4% of the male patients (93 of 136) and 77.3% of the female patients (99 of 128) in the average willingness group had poor glycemic control.

### 3.3. Diabetes Treatment Regimen

Method of diabetes therapy and level of glycemic control (*P* < 0.001) are noted in Table 2. Nearly 67.3% of males and 65.4% of females were treated with an oral agent, and 28.3% of males and 30.2% of females were treated with combination therapy. Interestingly, the same percentage of males and of females 4.4% were treated with insulin. Also, there was a positive association between types of medication and the degree of glycemic control in male (*P* < 0.001) and female (*P* < 0.001) patients (see Table 2).

### 3.4. Effects of Intervention

The information presented in Table 3 shows that the male patients in the program for a period of 6–10 years had a 0.01% decrease in their HbA1c level (7.60% at the most recent follow-up test minus 7.70% at the initial visit test; *P* = 0.300). There were no significant changes in HbA1c levels between the initial visit and the most recent follow-up visit in the program for the 0–5 years' group and in the male group. The female patients treated at the CSMUH diabetes unit for 6–10 years had a median increase of 0.05% in their HbA1c levels (8.05% at the most recent follow-up test minus 8.00% at the initial test; *P* = 0.670), compared with a decrease of 0.02% (7.60% at the most recent follow-up test minus 7.80% at the initial test; *P* = 0.196) in the patients treated for 0–5 years. The median change in the level of HbA1c in the female group was a decrease of 0.10%, but there were no statistically significant differences (*P* = 0.384).

### 3.5. Association between Predictive Factors and HbA1c Outcomes

Table 4 shows that a longer duration of diabetes (11–15 yrs) was positively associated with HbA1c outcomes in all patients (*B* = 1.147; *P* = 0.010) and in male patients (*B* = 2.281; *P* = 0.010), whereas a shorter duration of diabetes (0–5 yrs) was negatively associated with HbA1c outcomes in female patients (*B* = −0.519; *P* = 0.002). Use of oral therapy was negatively associated with HbA1c outcomes in female patients (*B* = −1.495; *P* = 0.009); use of insulin therapy was negatively associated with HbA1c outcomes in all patients (*B* = −1.032; *P* = 0.005) and female patients (*B* = −1.202; *P* = 0.000). Females' partial medication compliance (*B* = 0.318; *P* = 0.018), higher ages (45–54 yrs) (*B* = 0.455; *P* = 0.034), and junior high education level (*B* = 0.393; *P* = 0.039) were also positively associated with HbA1c outcomes.

## 4. Discussion

In this study, our aim was to investigate how the degree of glycemic control in patients with type 2 diabetes associates with lifestyle interventions as well as sociodemographic factors and further examine the differences by gender. The results of this study are intriguing and show that there appear to be sex-based differences in the stage and severity of diabetes. While further investigating the results of those out of the target range (HbA1c < 7.0%) and those with HbA1c ≥ 7.0% in terms of gender, in any subcategories of age, years of CSMUH, duration of diabetes, education level, family history, BMI, physical activity, smoking status, alcohol status, SMBG, medication compliance, self-management willingness, or type of medication, there were more percentages of female patients having an HbA1c level ≥ 7.0% than those of male patients. Some even reach significant differences, for instance, 0–5 years of CSMUH (*P* = 0.000), 6–10 years and >16 years of diabetes duration (*P* = 0.029; *P* = 0.009), elementary education level (*P* = 0.007), and BMI ≥ 25 and <25 kg/m^2^ (*P* = 0.010; *P* = 0.003). In addition, in any age subcategory (20–44; 45–54; 55–64; >65), more percentages of female patients (*P* = 0.038; *P* = 0.003; *P* = 0.034; and *P* = 0.046) significantly have an HbA1c level ≥ 7.0% than those of male patients.

The median HbA1c levels for female patients, whether they had been treated for 0–5 years or 6–10 years, and for the whole group, were all higher at the initial visit and the most recent visit than they were for men. This outcome indicates that the female patients were less likely to have good metabolic control than the male patients. The impact of this health inequality seems to be related to socioeconomic conditions. In our study, more women than men were from a poorer economic background. We found that that 27.3% of women and 8.4% of men in our study were illiterate.

In addition, diabetes is a particularly serious health condition in the aging population. A nationwide study found that late-middle-age and older Taiwanese women were on average less healthy than men of the same ages (13.7% versus 23.0%). The research data confirms that women between 45 and 54 years old tend to have increasing levels of HbA1c. In this study, we found that 41.9% of men and 44.5% of women only partially complied with their prescribed treatment regimens. This finding is consistent with the previous research, indicating that many diabetes patients partially comply with their treatment regimens, including both oral medication and insulin [12]. It is clear from this study that the results of disease self-management (regarding SMBG and self-management willingness) are similar regardless of the sex of the patient. Because of the complexity of medication options for type 2 diabetes and the heterogeneity of the disease process, treatment options should be individualized based on the needs and preferences of the prescriber and the patient [13]. Oral medications play an important role in the management of type 2 diabetes; insulin is often used after other treatments have failed. Some researchers have suggested that insulin is underused [14–16]. One study has addressed the complexity of DM regimens based on large national datasets: Koro and his colleagues, using National Health and Nutrition Examination Survey data from 1988 to 2000, showed that oral hypoglycemic agent use has increased from 45% to 53%, while insulin use has decreased from 24% to 16%. Combination therapy using both insulin and oral agents has increased from 3% to 11% [17]. Compared to the results of the study carried out by Koro and his colleagues, this paper reveals that patients are more likely to use oral hypoglycemic agents or combined therapy.

It is interesting to show that so few patients required insulin treatment over time. In order to investigate the interesting phenomenon, some medical papers were reviewed and clinicians were consulted. Type 2 diabetes was formerly known as non-insulin-dependent diabetes. Unlike type 1 diabetes, insulin injections are not initially required for type 2 diabetes treatment, for the early stage of type 2 diabetes is characterized by insulin overproduction. However, as the type 2 diabetes develops, the insulin secretion is becoming insufficient. Therefore, there comes to an international clinical consensus to recommend patients use metformin in combination with insulin in type 2 diabetes patients who initiate insulin treatment. For those having longer type 2 diabetes duration and being diagnosed the failure of insulin secretion, the clinicians may recommend the insulin injections alone [18–20]. Nonetheless, in patient-centered medication, patients in Taiwan are reluctant or have no preference to take insulin injections alone for long time use due to inconvenience and a proof of their failure in their diet control and weight control. The acceptable treatment for those HbA1c over 8 is always the combination of oral medication and insulin treatment. Indeed, approximately 66% of the patients used oral hypoglycemic agents (OHA); 29% used a combination of OHA and insulin, and 4% used insulin alone. In our study, we found that medication therapy with an insulin agent for type 2 diabetes was associated with a decreased level of HbA1c in all patients and in female patients. Therapy with an oral agent was associated with lower HbA1c levels in females. We looked at patients who had a first-degree relative with diabetes. Based on the information given by the patients, 54.4% of the male group and 52.4% of the female group had a first-degree relative with diabetes.

Examining the most recent follow-up results, the average HbA1c level was 7.70%, reduced from 7.80% in the female group. Based on the National Health and Nutrition Examination Survey data, the national mean HbA1c declined from 7.82% in 1999-2000 to 7.18% in 2004 [21]. Our study showed that 34.9% (*n* = 349) of the male patients and 27.5% (*n* = 281) of the female patients achieved the target level of <7.0%. In 2006, statistics showed that of the patients with type 2 diabetes in Taiwan, only 32.4% met the HbA1c < 7.0% target, and the average HbA1c level was 7.9% [22]. The National Health and Nutrition Examination Survey 1999-2000 data showed that 63.0% of people diagnosed with diabetes had an HbA1c level of >7.0%, with 37.0% having an HbA1c level of >8.0%, meaning that they had not met the target for the general population [23]. In subsequent years, the numbers have not improved significantly, with approximately 30.0% having an HbA1c level of >9.0% in 2004 and 2005 [24]. Nevertheless, in some studies, only 57.1% of adults diagnosed with diabetes achieved an HbA1c level of <7.0% [25].

The research results were based on the assumption that the patients had adhered to diet recommendations. When interpreting and generalizing the research results, those patients not adhering to diet recommendations should be excluded.

Changes in HbA1c reflect baseline A1c levels and individual patient characteristics such as age and general health status. Table 4 shows that female patients in 45–54 years' age group were also positively associated with HbA1c changes, but no association in overall and males group occurs. As shown in Table 2, the average age of the female diabetics patients (64.15) was significantly higher (*P* < 0.01) than that of the male patients (61.07). Also, the diagnosis age of type 2 diabetes in females (53.37) was significantly higher (*P* < 0.01) than that of males (51.42). Therefore, there came a difference of 1.14 years between male patients (10.34) and female patients (11.48). Though there was no significant difference (*P* = 0.61) in the years under the CSMUH plan between male and female patient, in the categories shown in Table 2, compared to male patients, more female patients were significantly out of the target range (HbA1c < 7.0%). Hence, it can be implied that age may serve as an important driver on effects of glycemic control. It could be possible that when the age is higher the metabolism is lowered or that there are different body structures or metabolism differences between males and females, hence causing different HbA1c outcomes. Also, as above mentioned, those less educated would lead to a lower socioeconomic status. Females were less well educated overall in this study population and were from a poorer economic background. Therefore, it can be suggested that socioeconomic and age differences between male and female populations played important roles causing more percentages of female patients to have an HbA1c level ≥ 7.0% than those of male patients.

## 5. Conclusions

The results of this study contribute to an understanding of how the degree of diabetes control and delivery of an ongoing diabetes self-management support program impact patient-reported outcomes. The findings also suggested that socioeconomic and age differences between male and female populations are more important drivers than any specific gender effects. Although management in all settings was suboptimal, the results pertaining to the male patients in the diabetes “pay-for-performance” plan indicate better glycemic control. This study also puts forward a few policy recommendations: the research results suggest that the development of innovative health promotion programs designed not only for the general population but also for each gender would be of value. Health inequality is associated with gender and socioeconomic status in Taiwan and is disease-specific. The results of these analyses provide important insights for policy makers to formulate healthcare policies related to chronic diseases or illnesses.

## Figures and Tables

**Table 1:** Characteristics of male and female patients in the year of study.

Characteristic	Overall	Male	Female	*P* ^a^
Age (years)	62.62	61.07	64.15	0.000
Years under the CSMUH plan	4.40	4.28	4.52	0.610
Age at diagnosis (years)	52.41	51.42	53.37	0.000
Diabetes duration (years)	10.91	10.34	11.48	0.002
BMI (<25 kg/m^2^)∗				
≥25 (%)	56.03	58.76	53.17	0.032
<25 (%)	43.97	41.24	46.83	

∗Initial visit level.

^a^Comparison by sex.

**Table 2:** Patient characteristics, self-management measurements, and diabetes therapy by gender at the most recent follow-up visit.

	Male													Female												
	HbA1c (%)													HbA1c (%)												
	<6.5		6.5–<7.0		7.0–8.0		>8.0		Sum		*χ* ^2^	df	*P*	<6.5		6.5–<7.0		7.0–8.0		>8.0		Sum		*χ* ^2^	df	*P*
	No.	(%)	No.	(%)	No.	(%)	No.	(%)	No.	(%)				No.	(%)	No.	(%)	No.	(%)	No.	(%)	No.	(%)			
Age group (yrs)											14.095	9	0.119											7.749	9	0.560
20–44	25	(29.8)	12	(14.3)	16	(19.0)	31	(36.9)	84	(8.4)				9	(16.4)	7	(12.7)	16	(29.1)	23	(41.8)	55	(5.4)			
45–54	33	(16.8)	35	(17.8)	57	(28.9)	72	(36.5)	197	(19.7)				14	(10.1)	15	(10.8)	45	(32.4)	65	(46.8)	139	(13.6)			
55–64	59	(17.6)	48	(14.3)	101	(30.1)	128	(38.1)	336	(33.6)				39	(12.5)	40	(12.8)	96	(30.8)	137	(43.9)	312	(30.5)			
>65	62	(6.2)	75	(19.6)	102	(26.6)	144	(37.6)	383	(38.3)				85	(16.5)	72	(14.0)	160	(31.0)	199	(38.6)	516	(50.5)			
Total	**179**	**(17.9)**	**170**	**(17.0)**	**276**	**(27.6)**	**375**	**(37.5)**	**1000**	**(100.0)**	****		****	**147**	**(14.4)**	**134**	**(13.1)**	**317**	**(31.0)**	**424**	**(41.5)**	**1022**	**(100.0)**			
Years of CSMUH care											21.056	3	<0.001											17.007	3	0.001
0–5	159	(20.7)	133	(17.3)	196	(25.6)	279	(36.4)	767	(76.7)				124	(16.0)	107	(13.8)	248	(32.0)	295	(38.1)	774	(75.7)			
6–10	20	(8.6)	37	(15.9)	80	(34.3)	96	(41.2)	233	(23.3)				23	(9.3)	27	(10.9)	69	(27.8)	127	(51.2)	248	(24.3)			
Total	**179**	**(17.9)**	**170**	**(17.0)**	**276**	**(27.6)**	**375**	**(37.5)**	**1000**	**(100.0)**	****		****	**147**	**(14.4)**	**134**	**(13.1)**	**317**	**(31.0)**	**424**	**(41.5)**	**1022**	**(100.0)**			
Duration of diabetes (yrs)											63.792	9	<0.001											74.161	9	<0.001
0–5	80	(32.0)	44	(17.6)	67	(26.8)	59	(23.6)	250	(25.0)				59	(25.1)	48	(20.4)	69	(29.4)	59	(25.1)	235	(23.3)			
6–10	53	(16.3)	59	(18.2)	92	(28.3)	121	(37.2)	325	(32.5)				39	(13.8)	38	(13.5)	94	(33.3)	111	(39.4)	282	(28.0)			
11–15	27	(11.8)	36	(15.7)	59	(25.8)	107	(46.7)	229	(22.9)				29	(11.7)	24	(9.7)	80	(32.3)	115	(46.4)	248	(24.6)			
≥16	16	(8.8)	30	(16.6)	52	(28.7)	83	(45.9)	181	(18.1)				16	(6.6)	23	(9.5)	69	(28.5)	134	(55.4)	242	(24.0)			
Total	**176**	**(17.6)**	**169**	**(16.9)**	**270**	**(27.0)**	**370**	**(37.0)**	**1000**	**(100.0)**	****		****	**143**	**(14.2)**	**133**	**(13.2)**	**312**	**(31.0)**	**419**	**(41.6)**	**1007**	**(100.0)**			
Education level											10.884	12	0.539											5.220	12	0.950
Illiteracy	12	(15.4)	12	(15.4)	19	(24.4)	35	(44.9)	78	(8.4)				38	(14.7)	38	(14.7)	82	(31.8)	100	(38.8)	258	(27.3)			
Elementary school	53	(17.7)	53	(17.7)	90	(30.0)	104	(34.7)	300	(32.3)				52	(14.5)	43	(12.0)	115	(32.1)	148	(41.3)	358	(37.9)			
Junior high	29	(19.3)	22	(14.7)	43	(28.7)	56	(37.3)	150	(16.1)				20	(14.9)	15	(11.2)	41	(30.6)	58	(43.3)	134	(14.2)			
Senior high	44	(18.7)	34	(14.5)	71	(30.2)	86	(36.6)	235	(25.3)				20	(14.6)	17	(12.4)	40	(29.2)	60	(43.8)	137	(14.5)			
College degree or higher	32	(19.2)	38	(22.8)	36	(21.6)	61	(36.5)	167	(18.0)				11	(19.0)	10	(17.2)	19	(32.8)	18	(31.0)	58	(6.1)			
Total	**170**	**(18.3)**	**159**	**(17.1)**	**259**	**(27.8)**	**342**	**(36.8)**	**930**	**(100.0)**	****		****	**141**	**(14.9)**	**123**	**(13.0)**	**297**	**(31.4)**	**384**	**(40.6)**	**945**	**(100.0)**			
Family history											0.283	3	0.963											7.558	3	0.056
No	76	(19.1)	70	(17.6)	106	(26.7)	145	(36.5)	397	(45.6)				76	(18.0)	55	(13.0)	132	(31.2)	160	(37.8)	423	(47.6)			
Yes	86	(18.2)	80	(16.9)	128	(27.1)	179	(37.8)	473	(54.4)				54	(11.6)	62	(13.3)	150	(32.3)	199	(42.8)	465	(52.4)			
Total	**162**	**(18.6)**	**150**	**(17.2)**	**234**	**(26.9)**	**324**	**(37.2)**	**870**	**(100.0)**	****		****	**130**	**(14.6)**	**117**	**(13.2)**	**282**	**(31.8)**	**359**	**(40.4)**	**888**	**(100.0)**			
BMI (<25 kg/m^2^)∗											13.353	6	0.038											5.189	6	0.520
≥25	84	(19.3)	74	(17.0)	118	(27.1)	160	(36.7)	436	(43.6)				55	(14.6)	53	(14.1)	115	(30.5)	154	(40.8)	377	(36.9)			
<25	64	(20.9)	59	(19.3)	77	(25.2)	106	(34.6)	306	(30.6)				50	(15.1)	49	(14.8)	105	(31.6)	128	(38.6)	332	(32.5)			
Unknown	31	(12.0)	37	(14.3)	81	(31.4)	109	(42.2)	258	(25.8)				42	(13.4)	32	(10.2)	97	(31.0)	142	(45.4)	313	(30.6)			
Total	**179**	**(17.9)**	**170**	**(17.0)**	**276**	**(27.6)**	**375**	**(37.5)**	**1000**	**(100.0)**				**147**	**(14.4)**	**134**	**(13.1)**	**317**	**(31.0)**	**424**	**(41.5)**	**1022**	**(100.0)**			
Physical activity (150 min/weekly)											14.103	3	0.003											7.706	3	0.052
No	113	(18.2)	90	(14.5)	162	(26.0)	257	(41.3)	622	(62.2)				82	(13.2)	77	(12.4)	182	(29.4)	278	(44.9)	619	(60.6)			
Yes	66	(17.5)	80	(21.2)	114	(30.2)	118	(31.2)	378	(37.8)				65	(16.1)	57	(14.1)	135	(33.5)	146	(36.2)	403	(39.4)			
Total	**179**	**(17.9)**	**170**	**(17.0)**	**276**	**(27.6)**	**375**	**(37.5)**	**1000**	**(100.0)**				**147**	**(14.4)**	**134**	**(13.1)**	**317**	**(31.0)**	**424**	**(41.5)**	**1022**	**(100.0)**			
Smoking status											1.745	3	0.627											1.491	3	0.684
No	137	(17.6)	134	(17.2)	218	(28.0)	289	(37.1)	778	(83.4)				139	(14.8)	120	(12.8)	293	(31.2)	386	(41.2)	938	(97.6)			
Yes	34	(21.9)	27	(17.4)	41	(26.5)	53	(34.2)	155	(16.6)				3	(13.0)	4	(17.4)	9	(39.1)	7	(30.4)	23	(2.4)			
Total	**171**	**(18.3)**	**161**	**(17.3)**	**259**	**(27.8)**	**342**	**(36.7)**	**933**	**(100.0)**	****		****	**142**	**(14.8)**	**124**	**(12.9)**	**302**	**(31.4)**	**393**	**(40.9)**	**961**	**(100.0)**			
Alcohol status											4.203	3	0.240											2.280	3	0.516
No	48	(17.1)	39	(13.9)	82	(29.2)	112	(39.9)	281	(29.9)				137	(14.6)	123	(13.1)	292	(31.1)	388	(41.3)	940	(96.8)			
Yes	124	(18.8)	122	(18.5)	180	(27.4)	232	(35.3)	658	(70.1)				5	(16.1)	4	(12.9)	13	(41.9)	9	(29.0)	31	(3.2)			
Total	**172**	**(18.3)**	**161**	**(17.1)**	**262**	**(27.9)**	**344**	**(36.6)**	**939**	**(100.0)**	****		****	**142**	**(14.6)**	**127**	**(13.1)**	**305**	**(31.4)**	**397**	**(40.9)**	**971**	**(100.0)**			
SMBG Once/daily											1.342	3	0.719											2.490	3	0.477
No	170	(17.9)	164	(17.3)	261	(27.5)	353	(37.2)	948	(94.8)				142	(14.7)	127	(13.2)	295	(30.6)	401	(41.6)	965	(94.4)			
Yes	9	(17.3)	6	(11.5)	15	(28.8)	22	(42.3)	52	(5.2)				5	(8.8)	7	(12.3)	22	(38.6)	23	(40.4)	57	(5.6)			
Total	**179**	**(17.9)**	**170**	**(17.0)**	**276**	**(27.6)**	**375**	**(37.5)**	**1000**	**(100.0)**				**147**	**(14.4)**	**134**	**(13.1)**	**317**	**(31.0)**	**424**	**(41.5)**	**1022**	**(100.0)**			
SMBG Once/weekly											5.919	3	0.116											5.430	3	0.143
No	73	(17.0)	79	(18.4)	131	(30.5)	147	(34.2)	430	(48.7)				85	(17.0)	70	(14.0)	152	(30.4)	193	(38.6)	500	(53.8)			
Yes	92	(20.3)	73	(16.1)	112	(24.7)	176	(38.9)	453	(51.3)				54	(12.5)	51	(11.8)	139	(32.3)	187	(43.4)	431	(46.2)			
Total	**165**	**(18.7)**	**152**	**(17.2)**	**243**	**(27.5)**	**323**	**(36.6)**	**883**	**(100.0)**	****		****	**139**	**(14.9)**	**121**	**(13.0)**	**290**	**(31.2)**	**380**	**(40.9)**	**930**	**(100.0)**			
Medication compliance											0.445	3	0.931											8.403	3	0.038
Full	83	(18.2)	85	(18.7)	122	(26.8)	165	(36.3)	455	(58.1)				79	(17.3)	73	(16.0)	142	(31.1)	162	(35.5)	456	(55.5)			
Partial	64	(19.5)	56	(17.1)	89	(27.1)	119	(36.3)	328	(41.9)				48	(13.2)	42	(11.5)	117	(32.1)	158	(43.3)	365	(44.5)			
Total	**147**	**(18.8)**	**141**	**(18.0)**	**211**	**(26.9)**	**284**	**(36.3)**	**783**	**(100.0)**	****		****	**127**	**(15.5)**	**115**	**(14.0)**	**259**	**(31.5)**	**320**	**(39.0)**	**821**	**(100.0)**			
Self-management willingness											7.088	3	0.069											7.290	3	0.063
Strong	122	(18.9)	123	(19.0)	180	(27.8)	222	(34.3)	647	(82.6)				108	(15.6)	105	(15.2)	221	(31.9)	259	(37.4)	693	(84.4)			
Average	25	(18.4)	18	(13.2)	31	(22.8)	62	(45.6)	136	(17.4)				19	(14.8)	10	(7.8)	38	(29.7)	61	(47.7)	128	(15.6)			
Total	**147**	**(18.8)**	**141**	**(18.0)**	**211**	**(26.9)**	**284**	**(36.3)**	**783**	**(100.0)**	****	****	****	**127**	**(15.5)**	**115**	**(14.0)**	**259**	**(31.5)**	**320**	**(39.0)**	**821**	**(100.0)**			
Categorized by type of medication											137.529	9	<0.001											120.758	9	<0.001
Oral medication(s) alone	151	(22.4)	144	(21.4)	203	(30.2)	176	(26.2)	673	(67.3)				129	(19.3)	107	(16.0)	231	(34.6)	201	(30.1)	668	(65.4)			
Insulin alone	8	(18.2)	8	(18.2)	10	(22.7)	18	(40.9)	44	(4.4)				5	(11.1)	6	(13.3)	9	(20.0)	25	(55.6)	45	(4.4)			
Both oral medication and insulin	20	(7.1)	18	(6.4)	64	(22.6)	181	(64.0)	283	(28.3)				13	(4.2)	21	(6.8)	77	(24.9)	198	(64.1)	309	(30.2)			
Total	**179**	**(17.9)**	**170**	**(17.0)**	**276**	**(27.6)**	**375**	**(37.5)**	**1000**	**(100.0)**				**147**	**(14.4)**	**134**	**(13.1)**	**317**	**(31.0)**	**424**	**(41.5)**	**1022**	**(100.0)**			

∗Initial visit level.

No.: Number.

df: degree of freedom.

**Table 3:** Effect of intervention on glycemic control by gender.

	No.	Years of CSMUH care		Median	Interquartile range	*P*	Age at diagnosis (years)
							Mean ± SD
Male	767	0–5	Initial visit HbA1c	7.50	1.05	0.677	51.48 ± 12.17
			Most recent visit HbA1c	7.50	1.10		
	233	6–10	Initial visit HbA1c	7.70	1.58	0.300	51.05 ± 11.17
			Most recent visit HbA1c	7.60	0.81		
	1,000	Total	Initial visit HbA1c	7.50	1.10	0.541	51.42 ± 12.04
			Most recent visit HbA1c	7.50	1.00		

Female	774	0–5	Initial visit HbA1c	7.80	1.00	0.196	53.63 ± 11.48
			Most recent visit HbA1c	7.60	1.05		
	248	6–10	Initial visit HbA1c	8.00	1.45	0.670	51.74 ± 9.70
			Most recent visit HbA1c	8.05	1.43		
	1,022	Total	Initial visit HbA1c	7.80	1.05	0.384	53.37 ± 11.26
			Most recent visit HbA1c	7.70	1.06		

No.: Number.

SD: standard deviation.

**Table 4:** Multiple linear regression between potential predictive factors and HbA1c outcomes in type 2 diabetic patients by gender.

Most recent visit HbA1c						
	Variable	*B*	Standard error	*P* value	95% CI	
					Lower	Upper
Overall	Constant	8.444	0.322	0.000	7.812	9.076
	Duration of diabetes					
	11–15 (yrs)	1.147	0.446	0.01	0.273	2.022
	Medication regimen					
	Insulin alone	−1.032	0.370	0.005	−1.758	−0.307

Male	Constant	7.637	0.360	0.000	6.929	8.345
	Duration of diabetes					
	11–15 (yrs)	2.281	0.879	0.010	0.554	4.008

Female	Constant	8.498	0.190	0.000	8.124	8.871
	Medication compliance	0.318	0.134	0.018	0.055	0.581
	Age group					
	45–54 (yrs)	0.455	0.214	0.034	0.035	0.874
	Duration of diabetes					
	0–5 (yrs)	−0.519	0.164	0.002	−0.842	−0.197
	Education level					
	Junior high	0.393	0.190	0.039	0.019	0.767
	Medication regimen					
	Oral alone	−1.495	0.574	0.009	−2.622	−0.368
	Insulin alone	−1.202	0.150	0.000	−1.498	−0.907

CI: confidence interval.
